# A calorimeter for analyzing ejected and non-ejected heat during Li-ion battery thermal runaway

**DOI:** 10.1016/j.isci.2025.112941

**Published:** 2025-06-18

**Authors:** Ola Willstrand, Mohit Pushp, Petra Andersson, Daniel Brandell

**Affiliations:** 1 RISE Research Institutes of Sweden, Box 857, 501 15 Borås, Sweden; 2 Department of Chemistry - Ångström Laboratory, Uppsala University, Box 358, 751 21 Uppsala, Sweden; 3 Division of Fire Safety Engineering, Lund University, Box 118, 221 00 Lund, Sweden

**Keywords:** Energy systems, Thermal engineering, Energy storage

## Abstract

Thermal runaway in lithium-ion battery cells poses significant safety risks due to rapid heat generation and potential thermal propagation within a battery system. This study investigates the total heat released and the fraction of energy contained in gas and particles ejected during thermal runaway using a purpose-built calorimeter setup. The results show that the fraction of ejected heat is significantly influenced by the state of charge (SOC) and cell mass loss. Notably, the non-ejected heat was higher at 75% SOC compared to 100% SOC due to higher fraction of ejected heat at high SOC. This will have implications in thermal propagation scenarios. Additionally, the study compares the results with accelerating rate calorimetry tests, highlighting the limitations of the latter in measuring the total heat released during thermal runaway. The findings show the need for comprehensive testing methods that can improve thermal management and safety in battery systems.

## Highlights

• Worst-case thermal propagation scenarios can occur at lower SOC levels than 100%
• Distinguishing between ejected and non-ejected heat is very important
• Method is proposed for evaluating heat and gas production during thermal runaway

## Graphical abstract

**Figure:**
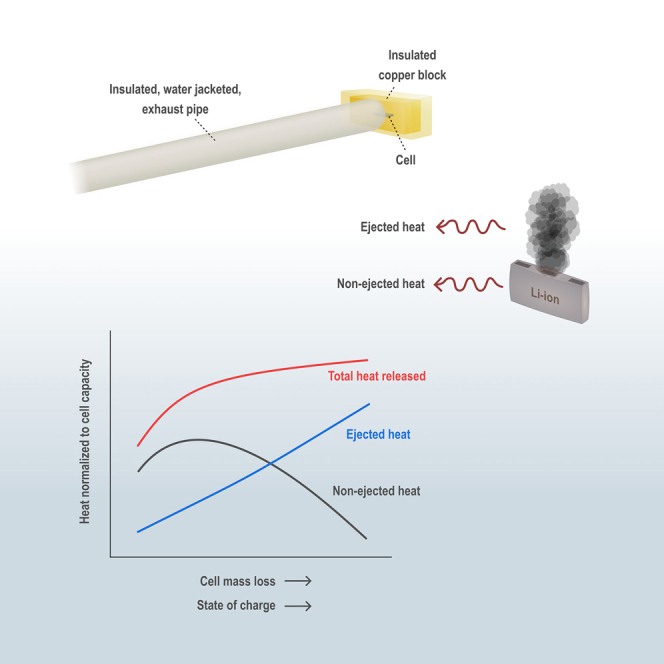

## Introduction

Battery safety is a highlighted area in society and thermal runaway (TR), characterized by a rapid and self-sustaining heat increase of the battery cell, is an important topic that needs to be fully understood and mitigated. TR of lithium-ion battery (LIB) cells may occur due to either mechanical, electrical, or thermal abuse, or in the absence of external triggers when an internal short circuit is induced due to e.g. a latent cell fault.^1^ TR in one cell may heat another cell until this cell also experiences TR, called thermal runaway propagation or simply thermal propagation (as defined in e.g. UN ECE regulations). In case heat is not sufficiently dissipated, thermal propagation may continue throughout the entire battery module or battery pack. A TR and thermal propagation may result in a battery fire, involving not only the cells but also electronics, cables, packaging material etc. Most of the heat from a battery fire is produced in the external combustion of ejected gases and particles, and combustion of surrounding material and components.^2^ This external combustion requires access to oxygen from surrounding air, and depending on the combustion efficiency, the total heat released (THR) from a battery fire is expected to be up to 5–10 times higher than the cell internal heat generation during TR.^3^ Thermal propagation over larger distances in air, e.g., between battery racks or between battery modules, will be aided by flaming fire. However, thermal propagation within a battery module or within a car battery pack, where battery cells are densely packed and the free air volume is limited, will instead be affected mainly by the cell internal heat generation. In this work, focus will be on the THR from LIB cells during TR in inert atmosphere, where no external combustion with air is taking place. Characterization of the heat release from single cells in inert atmosphere is important for comparison of different cells, as well as input to computer modeling of thermal propagation scenarios within battery modules and packs.

Accelerating rate calorimetry (ARC) is a commonly employed technique for studying thermal stability and heat generation in battery cells.^4^^,^^5^^,^^6^^,^^7^ It is also common to validate cell modeling of TR events with ARC tests.^8^^,^^9^^,^^10^ Test results typically include the cell temperature increase rate curve, as well as three characteristic temperatures: self-heating onset temperature (T_1_), TR onset temperature (T_2_), and cell maximum temperature (T_3_). The technique is focused on accurate measurements at low self-heating rates occurring before onset of TR, where adiabatic conditions are maintained in the test chamber. It is therefore a useful technique to determine the onset temperature of TR, which is an important parameter in thermal propagation modeling.^11^ On the other hand, the ARC chamber will not be able to retain adiabatic conditions during TR, and the heaters will be turned off to protect the test equipment. The maximum cell temperature recorded will therefore be affected by the non-adiabatic environment. This temperature, measured on the cell body, is also highly affected by the cell mass loss during TR. Despite that, several studies use this temperature to estimate the THR during TR.^8^^,^^12^^,^^13^^,^^14^^,^^15^ THR ratios of 0.7*E*-0.9*E*, 0.6*E*-1.6*E*, 0.7*E*-0.8*E*, 0.8*E*-1.4*E* (2.0*E*-3.3*E* when temperature increase of the test chamber was included), and 0.4*E*-0.5*E* respectively, where *E* is the available electrical energy in the cell, were obtained in these studies. It is questionable whether measurements of <1*E* are true THR, because no electrical energy is typically left after TR.

Other techniques that have been used to measure internal heat generation (sometimes referred to as the THR and sometimes not) include copper slug battery calorimetry (CSBC),^16^^,^^17^^,^^18^ bomb calorimetry,^19^ as well as other enthalpy change methods.^20^^,^^21^^,^^22^ Bomb calorimetry is a useful technique to measure the THR, because nothing leaves the calorimeter chamber in contrast to ARC and most other techniques. Lyon and Walters^19^ obtained a THR of 1.6*E*-2.8*E* at 50%–100% state of charge (SOC) using bomb calorimetry on four different cells. In general, a higher SOC will result in a higher THR but lower energy factor in relation to the available electrical energy in the cell, meaning that >2*E* was measured when *E* is low and <2*E* was measured in case of 100% SOC. However, bomb calorimetry is typically limited to small samples and small LIB cells because of the massive pressure build-up caused by TR in larger cells. CSBC, ARC, and the other enthalpy change methods, where the produced gases are released outside of the calorimeter, do not suffer from this limitation. But release of gases could potentially result in uncontrolled heat losses from the system, thereby underestimating the THR. The reported THR is also generally lower, e.g., 0.6*E*-1.3*E* (100% SOC) for CSBC,^16^^,^^17^^,^^18^ and 0.4*E*-0.8*E* for some other methods,^21^^,^^22^ which could indicate that not all produced heat was measured. In contrast, the method Said et al.^20^ have used, where the enthalpy change for an air stream is measured, which both is cooling the cell body and collecting the hot generated gases, resulted in THR of 1.6*E*-1.7*E* for different LIB cells. This is in line with the results from Lyon and Walters.^19^

All the previous studies and methods lack information about the fraction of ejected heat from the cell during TR, meaning ejected hot gases and hot particles. Sometimes, even a large part of the cell internal jellyroll is ejected during TR. Ejected mass will to a lesser extent contribute to the temperature increase of the cell casing and conductive heat transfer from the cell body to surrounding cells. For accurate thermal propagation modeling, it is important to separate the non-ejected cell body heat from the ejected heat. The ejected heat could potentially spread much larger distances and contribute to complete module or pack temperature increase, while the cell body conductive heat transfer will contribute to the temperature increase of the closest cells to a greater extent. For this purpose, NASA has developed a fractional thermal runaway calorimeter (FTRC), which can distinguish between ejected and non-ejected heat release from cylindrical LIB cells.^23^ Test data from hundreds of tests conducted in this apparatus are publicly available in the Battery Failure Databank.^24^ At 100% SOC, the THR varies between 0.9*E*-2.1*E* for 23 different small format cylindrical cells.^25^ Most of these cells ejected about 50%–85% of the original cell mass, where ejected mass carried more than ten times more heat per gram in relation to non-ejected mass. This shows the importance of knowing the different paths of heat transfer in a thermal propagation scenario inside a battery pack or module. To date, the Battery Failure Databank contains data only on small format cylindrical cells, even though work has been done to develop a large format FTRC.^26^

In this study, we have developed a battery calorimeter, making use of a copper block (CB), similar to the CSBC setup, which is connected to an exhaust pipe collecting and measuring the energy from ejected gases and solid mass. The setup is adoptable to a large range of cell sizes. Except measuring THR and fraction of ejected heat, we have also explored the possibility of measuring total gas production, gas composition, and gas production rate in the same setup. Here, we utilize this instrumentation to explore TR characteristics of small cylindrical cells as well as large format prismatic cells. In addition, the impact on a thermal propagation scenario from differences in amount of ejected heat is discussed, as well as the meaning of THR gained from ARC tests.

## Results

17 battery cell tests were conducted in the developed CB calorimeter. The cell type, capacity, and SOC for the different tests are presented in Table 1. Two different cylindrical 21700 cells from two different manufacturers were tested, including high-nickel NMC (cell A) and NCA (cell B) cathode materials. The two prismatic cells investigated were of similar type (NMC811), where prismatic cell D is an earlier generation of cell C, approximately one-third in size and capacity. Four different SOC levels were tested for the large format prismatic cell, and two different SOC levels for the cylindrical cells, as seen in Table 1.

**Table 1:** Test schedule for copper block calorimeter

Test	Cell type	Capacity	Cell Weight	SOC
1	Cyl. cell A	5 Ah	68 g	100%
2	Cyl. cell A	5 Ah	68 g	100%
3	Cyl. cell B	5 Ah	66 g	100%
4	Cyl. cell B	5 Ah	66 g	100%
5	Cyl. cell A	5 Ah	68 g	100%
6	Cyl. cell A	5 Ah	68 g	75%
7	Cyl. cell B	5 Ah	66 g	75%
8	Cyl. cell B	5 Ah	66 g	100%
9	Pris. cell C	157 Ah	2.1 kg	50%
10	Pris. cell C	157 Ah	2.1 kg	50%
11	Pris. cell C	157 Ah	2.1 kg	75%
12	Pris. cell C	157 Ah	2.1 kg	100%
13	Pris. cell C	157 Ah	2.1 kg	25%
14	Pris. cell C	157 Ah	2.1 kg	100%
15	Pris. cell C	157 Ah	2.1 kg	50%
16	Pris. cell C	157 Ah	2.1 kg	100%
17	Pris. cell D	55 Ah	0.84 kg	100%

### Copper block calorimeter

**Figure 1:**
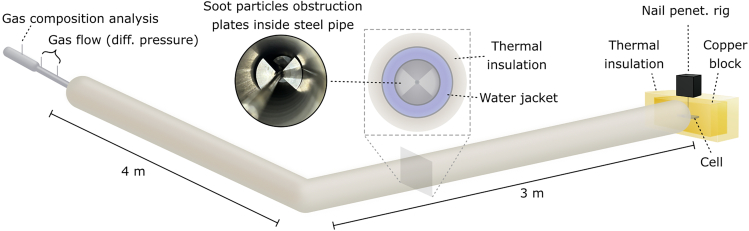
Calorimeter setup Copper block calorimeter. Cell is mounted in copper block to the right and heat from ejected hot particles and gases are measured in the insulated, water jacketed, exhaust pipe. Separate pipes are used for gas flow and gas composition measurements to the left in the figure. A photo of the setup is shown in Figure S1.

The CB test setup is illustrated in Figure 1. More details are found in the STAR Methods description. The CB was fully insulated, except for an outlet for gases and ejected particles in front of the cell’s safety vent, connected to the exhaust pipe. TR was initiated by nail penetration in all tests.

The exhaust line consisted of two fully insulated separate pipes, 3 m and 4 m each, where only the 3 m pipe was used for the smaller cylindrical cells. Each pipe had soot particles obstruction plates inside, seen in Figure 1, and a water jacket acting as a heat sink for the ejected particles and gases.

After the insulated pipes, the exhaust gas was guided through another pipe for gas flow measurements and gas composition analysis before released into the exhaust gas cleaning system of the test laboratory. The gas composition analysis was used to estimate the gas density in the gas flow calculations, as well as the gas specific heat capacity used to calculate the convective heat leaving the system.

In the CB test setup, the THR ($Q_{tot}$) is calculated according to:

(Equation 1) $$Q_{tot}={\left(c_{p}m\Delta T\right)}_{CB}+{\left(c_{p}m\Delta T\right)}_{cell}+{\left(c_{p}m\Delta T\right)}_{water}+{\left(c_{p}m\Delta T\right)}_{pipe}+Q_{gas}+Q_{loss}$$

where $c_{p}$ is the specific heat capacity, m is the mass, and ΔT is the temperature rise of the CB, the cell, the water, and the insulated pipes, respectively. Note that $m_{cell}$ is the mass of the cell after the test. Further, $Q_{gas}$ is the integrated convective heat flow measured directly after the insulated pipes:

(Equation 2) $$Q_{gas}=\int {\left(c_{p}\dot{m}\Delta T\right)}_{gas}$$

Finally, $Q_{loss}$ is the total conductive and radiative heat loss from the calorimeter system from start of TR until heat is evenly distributed in the system, i.e., when the cell temperature is the same as the average CB temperature; a period of up to 50 min for the large prismatic cell. The total heat loss is estimated based on the temperature decrease rate of the system after the heat has been evenly distributed, and with the assumption that the heat loss rate was constant during the test. This is a simplification since heat loss will vary with temperature, but within this time frame the difference between the average system temperature and the surrounding temperature did not change significantly (typically <1%). Note that the nail, used to penetrate the battery cell, might initially have caused a higher heat loss rate from the system due to a higher cell temperature as compared to the average system temperature. However, nail cross-sectional area is only 0.2% of the cylindrical cell surface area and only 0.02% of the large prismatic cell surface area, limiting this effect. In the performed test series, the total heat loss was estimated to approximately 3%–4% of the THR.

### Heat generation

**Figure 2:**
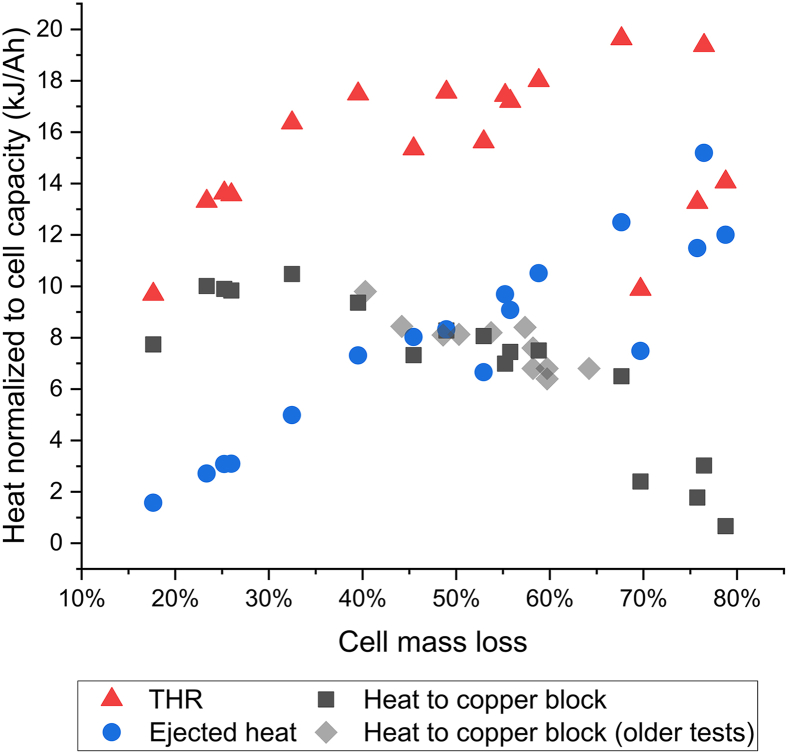
Heat generation as a function of cell mass loss Total heat released, ejected heat, and heat transferred to the copper block, divided by the rated capacity of the cell, as a function of cell mass loss.

The THR $\left(Q_{tot}\right)$, heat transferred to the CB ($Q_{CB})$, and the ejected heat ($Q_{gas}+Q_{water}+Q_{pipe}$), divided by the rated capacity of the cell, are shown as a function of cell mass loss in Figure 2 for all conducted tests. In addition, results from a previous test series with the same type of cells, where only the CB was used without measuring the ejected heat, is added for comparison, showing repeatability of the method. The large variation in cell mass loss is due to both variance in SOC and variance in cell type. The results show that the ejected heat as well as the non-ejected heat is strongly dependent on the cell mass loss. The higher the cell mass loss, the higher is the ejected heat and the lower is the heat transferred to the CB. In addition, heat generation normalized to rated cell capacity show similar results despite large variance in capacity. Cell capacity (for similar cell chemistries) is correlated to the amount of active electrode material, and thereby to cell size.

**Figure 3:**
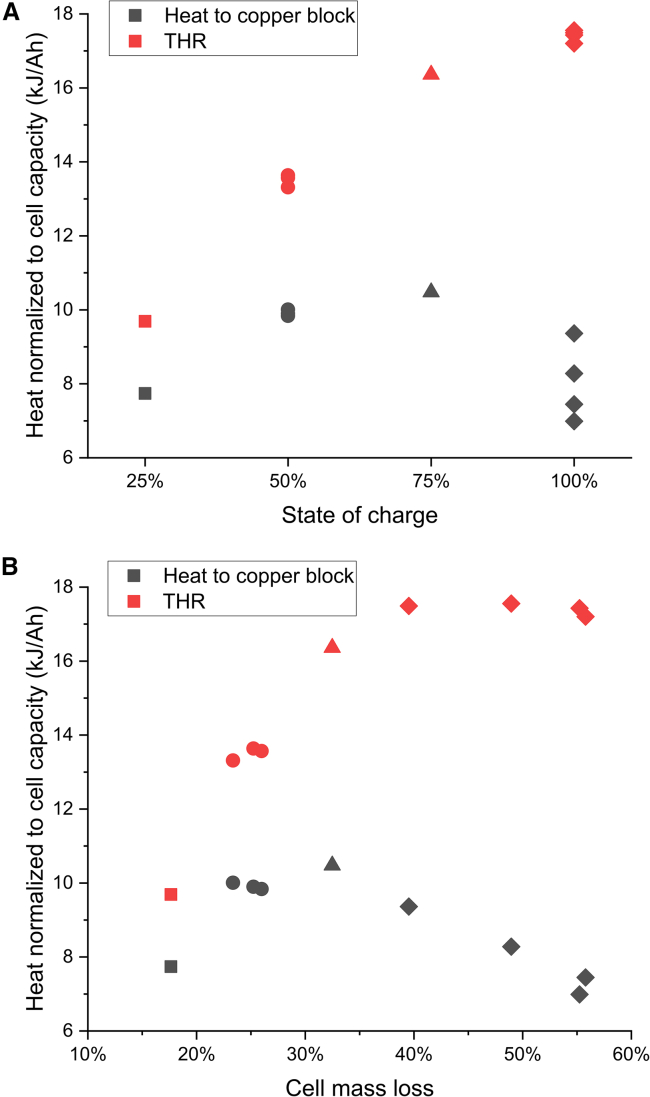
Heat generation for different SOC Heat transferred to the copper block, as well as THR, for the prismatic cells, as a function of (A) SOC, and (B) cell mass loss. The different symbols represent the four different SOC levels for easier identification of SOC level in (B).

To better see the dependence on the SOC, Figure 3 is showing results only from the prismatic cells. The THR increases with SOC, while the heat transferred to the CB seems to peak at 75% SOC, as seen in Figure 3A. Typically, higher SOC will result in a more rapid temperature increase of the cell and more violent TR scenario, resulting in higher cell mass loss. Therefore, the results are similar if presented as a function of cell mass loss, as in Figure 3B. However, the mass loss can vary, especially at high SOC, resulting in quite large differences in the heat transferred to the CB at 100% SOC, as seen for the diamond symbols in Figure 3B.

**Figure 4:**
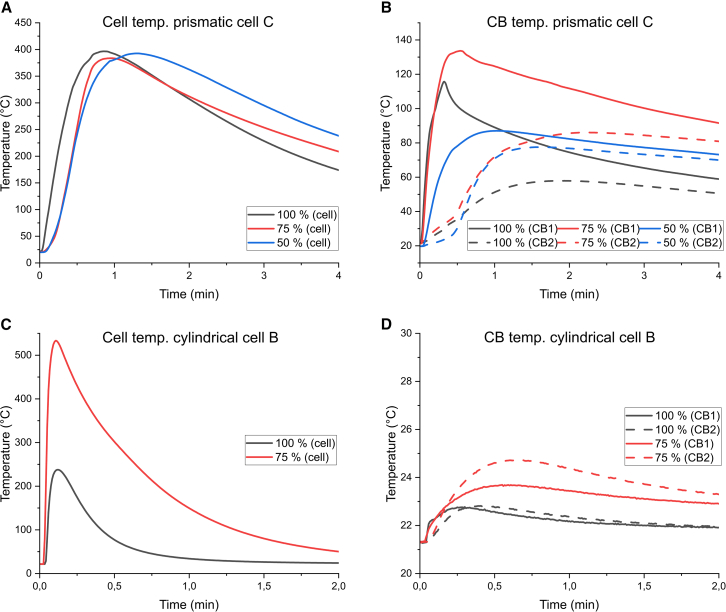
Temperature as a function of time Temperature as a function of time for selected tests at different SOC levels. (A) cell temperature of prismatic cell C (negative terminal), (B) two copper block temperatures positioned closest to prismatic cell C, (C) cell temperature of cylindrical cell B (cell lid), and (D) two copper block temperatures positioned closest to cylindrical cell B. See Figure S2 for thermocouple positions.

In Figure 3, it is seen that the largest amount of energy transferred to the CB is achieved at 75% SOC for the large prismatic cell, and even at 50% SOC the heat transferred to the CB is larger than at 100% SOC. This may have implications in a thermal propagation scenario, depending on whether the ejected or non-ejected heat is most detrimental. However, for the non-ejected heat (heat transferred to the CB), high heating power could be more critical for thermal propagation than the total energy transferred. In Figure 4A, it is shown that the initial temperature increase rate of the cell is highest at 100% SOC, indicating a higher heating power, but this is just for a short period and the maximum cell temperature reached is similar in all three tests. In addition, the temperature increase rate of the CB, shown in Figure 4B for the two thermocouples closest to the cell (CB1 and CB2), is similar both in case of 75% SOC and 100% SOC, but continues for longer time and reaches higher maximum temperatures in case of 75% SOC. See Figure S2 for thermocouple positions. At 50% SOC, the temperature increase rate is lower as compared to the higher SOC levels. Figures 4C and 4D shows the same trend for one of the cylindrical cells when comparing tests at 75% SOC and 100% SOC. The much lower maximum cell temperature for the test at 100% SOC is due to that most of the internal jellyroll was ejected.

**Figure 5:**
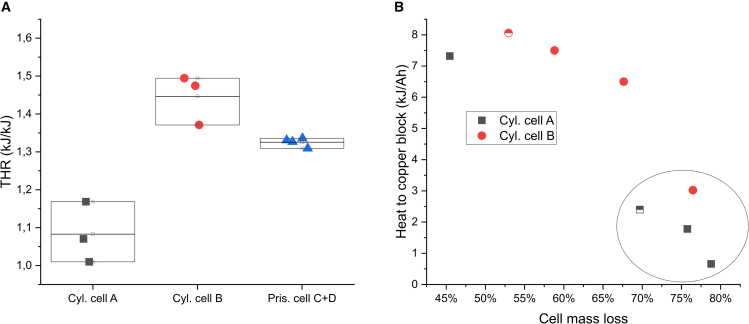
Heat generation for different cell type (A) THR normalized to available electrical energy in the different cells (at 100% SOC), and (B) heat transferred to the copper block for the two different cylindrical cells. Different colored symbols represent the different cell types. Filled symbols: 100% SOC, half-filled symbols: 75% SOC. The large circle in (B) highlights tests where the cell lid was ejected.

The THR normalized to cell capacity were similar for the different cells as shown in Figure 2. However, if sorting the results by cell type and considering only 100% SOC, significant differences between the tested cells are revealed, as seen in Figure 5A. Note that the normalization to available electrical energy instead of rated cell capacity, at a certain SOC level, is just a scaling factor since the voltage range is similar for all tested cells. There could be many reasons for the difference in THR between the different cells, including cell design and materials. The repeatability in the measured THR is very good for the prismatic cells. The larger variance for the cylindrical cells is probably due to the larger mass ratio between the setup and the cells, increasing the uncertainty. Despite adjustment of the masses of CB and exhaust pipe, the mass ratio between setup and the cells was a factor 10 larger for the cylindrical cells. Focusing on the two different types of cylindrical cells tested, which display significant difference in THR, it can be concluded that the amount of heat transferred to the CB is not significantly affected by neither cell type nor SOC, but only on the percentage of cell mass loss, seen in Figure 5B. The encircled samples in Figure 5B, having the largest mass loss and significantly lower amount of heat transferred to the CB, have all in common that the cell lid was ejected during the tests. For all other tests, the lid was still attached to the cell can, which means that gases and particles were ejected through dedicated vent channels through the lid. The ejection of the cell lid, affecting the total cell mass loss, happened for both cell A and B, but more frequent for cell A.

### Gas generation

**Figure 6:**
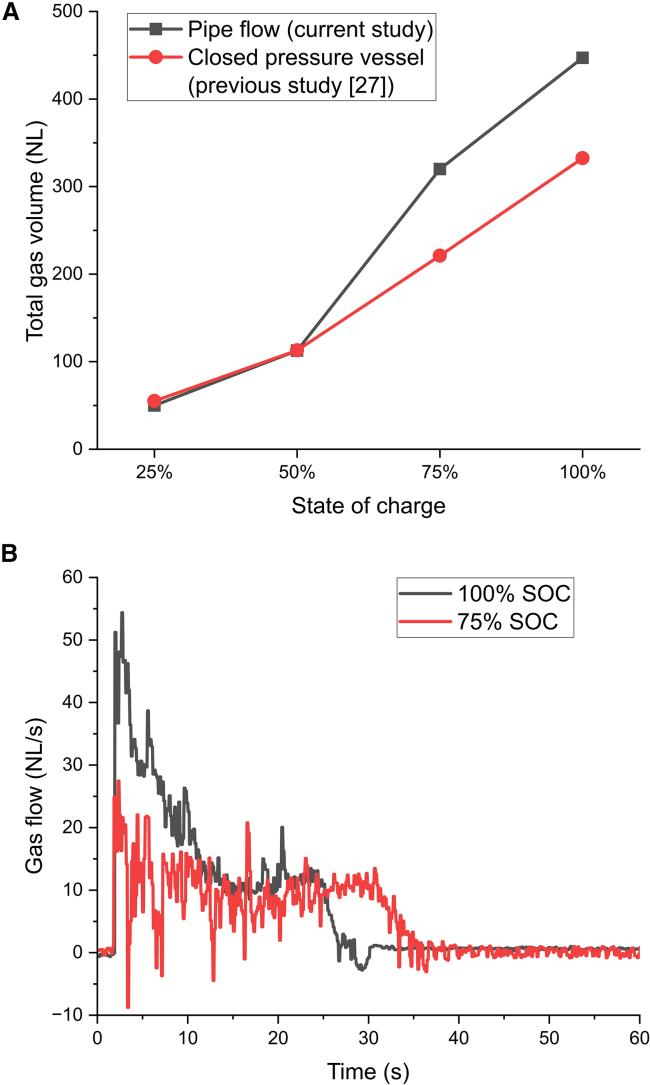
Gas generation (A) Total gas volume in normal liters (NL) at 1 atm and 20°C, and (B) normal gas flow for prismatic cell C at different SOC levels. See Figure S3 for gas flow at 50% SOC.

The total gas production for prismatic cell C at normal temperature and pressure (NTP) is presented in Figure 6A. The total gas volume is calculated by integrating the volume flow. This cell has previously been tested in a closed pressure vessel,^27^ where the total gas volume is calculated based on temperature and pressure rise inside the vessel, and these results are included in the figure for comparison. In the closed pressure vessel, the total gas volume is calculated when the pressure and temperature are stabilized below water dew point. In the measurement of the gas flow in the current study, the temperature of the gases was higher than in the closed pressure vessel, at least in tests with high SOC. The larger gas volume measured at high SOC in this study is probably due to that condensable substances may have been present in the gas phase. Note that the convective heat flow ($Q_{gas}$) only constitutes maximum 3% of the THR for the largest cell (cell C) when measured after the long water jacketed pipes cooling the gases. For the smaller cells, the convective heat flow is negligible.

The benefit of measuring the gas flow, rather than pressure build-up in a closed chamber, is that the gas production rate can be obtained more accurately. The gas flow for prismatic cell C at 75% and 100% SOC is shown in Figure 6B. A gas production period of approximately 30 s is observed, which is in good agreement with visual observation of tests with this cell in open atmosphere. The higher total gas production at 100% SOC can be attributed to the higher gas production rate the first 10–15 s. Another observation is the slightly longer period of gas production in case of 75% SOC, which might be caused by the lower cell mass loss that result in more material being kept at higher temperatures inside the cell. However, at even lower cell mass loss, as in case of 50% SOC, the period of gas production was similar to 100% SOC, see Figure S3, indicating that mass loss is not the only criteria but rather the amount of non-ejected heat which was greatest at 75% SOC (see Figure 3A).

The total gas production is typically dependent on SOC as showed for the prismatic cell and variation in cell mass loss for the same SOC level had low impact on the total gas production. However, this was not the case for the cylindrical cells tested at 75% and 100% SOC. In fact, the total gas production was similar for both cell A and B at both SOC levels, and only dependent on whether the cell lid was still in place or not. The encircled tests in Figure 5B had, on average, a total gas production that was 27% lower than the non-encircled tests. Apparently, the lack of the cell lid afterward indicates that solid material and/or liquid electrolyte were ejected to a greater extent, interrupting some of the gas producing reactions.

### Accelerating rate calorimetry

**Figure 7:**
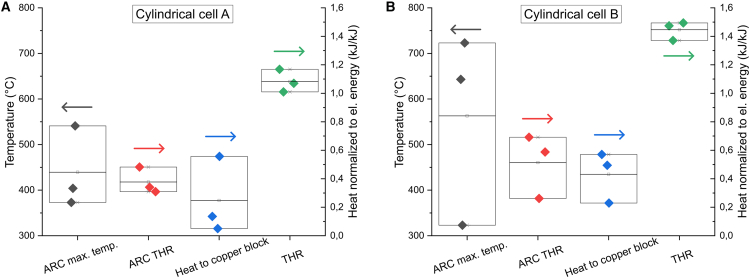
Comparison with ARC results ARC test results, in comparison with results from the copper block setup, for (A) cylindrical cell A, and (B) cylindrical cell B. Arrows shows which axis to read. Figure S4 shows complementary ARC results.

The two cylindrical cells tested with the CB calorimeter were also tested using a standard ARC equipment for comparison. Six tests were conducted, three of each cell type and all at 100% SOC. The maximum cell temperatures and the normalized THR, calculated based on the temperature rise of the cell, is shown in Figure 7 for the six ARC tests performed. In addition, heat transferred to the CB as well as THR obtained from the CB setup, for the same type of cells at 100% SOC, is included in the figure for comparison. In the ARC tests, the cell maximum temperatures varied significantly, thereby also affecting the calculated ARC THR. For cylindrical cell B, the variation of the ARC THR is similar to the variation in the heat transferred to the CB for the same type of cell, indicating that the ARC THR is dependent on the cell mass loss. From Figure 7, it is obvious that ARC THR neglects the ejected heat. However, in ARC THR calculations, the original cell mass is typically used, so also here, with the assumption that ejected mass and non-ejected mass reaches the same maximum temperature. In theory, it is therefore the actual THR that is calculated, but the results correlate only with the non-ejected heat, why those results are not reliable. ARC tests are therefore primarily meaningful to estimate the heat production before onset of TR, where adiabatic conditions are maintained in the test chamber. The self-heating onset temperature, the first venting temperature, and the TR onset temperature were thus all much more repeatable than the maximum cell temperature in the tests performed (see Figure S4).

## Discussion

The THR obtained for the different cells tested, 1.0*E*-1.5*E* at 100% SOC, and 1.0*E*-2.9*E* for all SOC levels, are expected based on available literature. Results less than 1.0*E* for the THR, frequently found in the literature, are probably not covering both non-ejected and ejected heat from the cell during TR, as seen in the conducted ARC tests.

Separating the ejected heat from the non-ejected heat has potentially great importance in the safety evaluation of battery systems. The results show that the total amount of heat transferred conductively from the cell body could be higher at both 75% and 50% SOC than at 100% SOC due to the higher cell mass loss at higher SOC and thereby larger amount of ejected heat. In addition, at 75% SOC the conductive power and the temperature increase rate of the surrounding CB were not lower than for 100% SOC. Almost all battery safety tests are conducted at 100% SOC, both standardized tests and other battery safety tests, based on in-house testing experience. This is generally considered the worst possible case, but the results in this study indicate that worst case thermal propagation scenarios might occur at slightly lower SOC levels, if the cell mass loss is greatly affected. For cylindrical cells, it was shown that the robustness and adherence of the cell lid during TR had a large impact on the cell mass loss and the amount of ejected heat, potentially detrimental for thermal propagation. This is also something discussed by Buckwell et al.,^28^ who concluded that a less robust lid may facilitate ejection of material, prevent cell case rupture, and limit the risk of thermal propagation. Note that the TR onset temperature is typically lower with increased SOC, and the highest SOC could therefore still be the worst-case scenario despite slightly lower conductive heat transfer. It also matters where the ejected heat is guided. This needs further investigation with actual thermal propagation tests on battery systems.

It is beneficial with test methods that include all relevant measurements, in this case both heat and gas generation. The setup could be further adapted such that either fully condensed or fully uncondensed flow is measured. In this test series, the gas flow from the largest cell was only partly condensed. The measured total gas volume at 75% and 100% SOC were 30%–40% higher than the expected condensed total gas volume. However, the difference might be even larger if measuring uncondensed gas flow, if considering that the produced molar quantity of water could be similar to the produced CO/CO_2_ content; in this case 60%–70% of the dry gases. This assumes that most of the CO/CO_2_ and water originates from decomposition of the electrolyte reacting with released oxygen from the cathode metal oxide.^29^^,^^30^ Other reaction pathways could result in other relative concentrations of CO/CO_2_ and water. Gas temperature and gas flow could be measured closer or further away from the cell to catch either fully condensed or uncondensed flow.

Overall, the test setup shows promising results. 97%–100% of the heat is initially transferred to either the CB, the water, or the metal pipes, and the total heat losses from the system during approximately one hour is only 3%–4% of the THR. The size of the CB, the length and weight of the pipes, the amount and flow rate of water, etc. can be optimized based on the tested cell, but this test series shows that three greatly varying cell sizes can be tested with just minor adjustments.

### Conclusions

This study utilized a purpose-built battery calorimeter setup, which included a CB connected to an exhaust pipe to measure the THR as well as the energy fraction from ejected gases and solid mass. The setup allowed for the assessment of various cell sizes, providing a versatile tool for the characterization of TR in LIB cells.

The results indicate that the THR are significantly influenced by the SOC and the cell size. The amount of ejected heat is mainly dependent on the cell mass loss during TR. Higher SOC levels generally result in greater cell mass loss and larger amount of ejected heat. Therefore, it is found that the total energy transferred to the CB was higher at 75% SOC compared to 100% SOC, suggesting that worst-case thermal propagation scenarios might occur at slightly lower SOC levels than 100%.

Separating the ejected heat from the non-ejected heat is important for understanding thermal propagation in battery systems and for accurate modeling thereof. The ejected heat, which includes hot gases and particles, can spread over larger distances and contribute to the overall temperature increase of a battery module or pack. In contrast, the conductive heat transfer from the cell body primarily affects the closest cells. This distinction is crucial for developing effective thermal management strategies in battery systems.

This study also compared the results obtained from the CB setup with those from ARC tests. The ARC tests, which are commonly used to study thermal stability and heat generation in battery cells, were found to underestimate the THR as they do not account for the ejected heat. The results underscore the limitations of ARC tests in capturing the full extent of heat generation during TR.

Furthermore, it was demonstrated that total gas production and gas production rate during TR can be measured using the same test setup. The total gas volume and production rate are dependent on the SOC and to some degree also on the cell mass loss, especially in case of high percentage of mass loss, as was the case when the cell lid on the cylindrical cells was ejected.

This calorimeter setup provides valuable insights into the thermal behavior of LIB cells during TR by offering a more accurate measurement of THR and ejected energy. These parameters are critical for improving thermal management and safety in battery systems. The findings thus emphasize the need for comprehensive testing methods that account for both ejected and non-ejected heat to better predict and mitigate thermal propagation scenarios.

### Limitations of the study

The developed calorimeter is a prototype which can be further improved based on extended test series. Especially, this study does not investigate e.g., pouch format cells or cells with lower energy density, such as LFP type cells. Furthermore, the study did not include thermal propagation tests to confirm the indicative results on the influence of cell mass loss and fraction of ejected heat. Note that the ejected heat is measured under inert conditions, excluding external combustion which could significantly increase the THR.

## Resource availability

### Lead contact

Requests for further information and resources should be directed to and will be fulfilled by the lead contact, Ola Willstrand (ola.willstrand@ri.se).

### Materials availability

This study did not generate new materials.

### Data and code availability

• All data reported in this paper will be shared by the lead contact upon request.
• This paper does not report original code.

## Acknowledgments

Abdilbari Shifa Mussa and Northvolt are acknowledged for performing the ARC tests. The work is part of a project funded by the 10.13039/501100004527Swedish Energy Agency (project no. 51787-1). Partners within the project comprise of RISE Research Institutes of Sweden, Northvolt, Scania, and Uppsala University. We also acknowledge support from the COFFEE project (funded by the 10.13039/501100007430Swedish Mercantile Marine Foundation and the 10.13039/501100013178Swedish Transport Administration), Batteries Sweden (10.13039/501100001858VINNOVA, grant no. 2019-00064 and 2024-03853), and the StandUp for Energy consortium.

## Author contributions

O.W.: conceptualization, methodology, formal analysis, investigation, writing – original draft. M.P.: methodology, writing – review and editing. P.A.: conceptualization, writing – review and editing, supervision. D.B.: conceptualization, writing – review and editing, supervision.

## Declaration of interests

The authors declare no competing interests.

## STAR★Methods

### Key resources table

**Table:** 

REAGENT or RESOURCE	SOURCE	IDENTIFIER
**Other**		

ARC	Thermal Hazard Technology (THT)	www.thermalhazardtechnology.com/battery-products/accelerating-rate-calorimeter
FCO432	Furness Controls	www.furness.com/FCO432/
HY-ALERTA 500	H2scan	h2scan.com/
X-STREAM XEGK (IR/IR/PO2)	Emerson	www.emerson.com/en-us/catalog/automation-solutions/measurement-instrumentation/gas-analysis/rosemount-x-stream-enhanced-xegk-continuous-gas-analyzer
BINOS 100 (CO/CH4)	Rosemount (Emerson)	www.emerson.com/documents/automation/manual-binos-100-series-analyzers-including-oxynos-100-hydros-100-cat-100-2nd-ed-rosemount-en-69714.pdf

### Method details

The calorimeter built for this study consisted of a copper block connected to an exhaust pipe as described in the results.

#### Copper block

The size of the copper block could be adjusted to match the tested cell. In the tests performed, the copper block mass was 77 kg for the cylindrical cells and 172-175 kg for the prismatic cells. Temperature monitoring of the copper block was conducted using 10-12 type K thermocouples, which were placed in 2.5 cm deep pre-drilled holes around the copper block and coated with heat transfer paste. One thermocouple was attached to the cell’s negative terminal to monitor the cell temperature. TR was initiated by nail penetration, using a pneumatically operated steel nail, 4 mm in diameter, rapidly injected into the centre of the cell’s side wall. The penetration depth was ensured to be at least half of the cell’s thickness. The nail penetration rig was positioned outside of the insulation, with the nail fed through a pre-drilled hole in the copper block. To determine the cell mass loss the battery cell was weighed before and after each test using a scale.

#### Exhaust pipe

The total weight of the insulated steel pipes was either 36 kg (1 pipe) or 68 kg (2 pipes), and the total weight of water was 15 kg (1 pipe) or 35 kg (2 pipes). Note that the higher weight of the shorter steel pipe is due to inclusion of pipe connections in both ends. 10-17 type K thermocouples were used to monitor the temperature distribution and temperature increase of the water and the steel pipes during the tests. In each test, the exhaust pipes were purged with nitrogen gas to create an inert atmosphere. To restore test conditions, the soot particle obstruction plates were removed from the pipe and cleaned after each test, and remaining particulate matter inside the pipe was vacuumed.

During TR, ejected heat from the cell will be unevenly distributed in the insulated pipes. For effective heat distribution, the water was circulated using a small pump(s) (see Figure S1). The heat generation due to work done by the water pump was subtracted from the THR calculations. See Figure S5 for an example of the corrected temperature increase of the water in one of the pipes.

#### Gas flow and composition analysis

Prior to tests, the smaller gas flow pipe used for the cylindrical cells was calibrated against a known air flow of 3.0-12.6 L/s and the larger pipe used for the prismatic cells against an air flow of 9.7-62.0 L/s. The volume flow ($\dot{V}$) was calculated according to:

$$\dot{V}=A{\left(\Delta p/\rho \right)}^{B}$$

where A and B are calibration constants, Δp is the differential pressure (measured using a *FCO432 ±500 Pa* instrument), and ρ is the gas density. In the tests, gases were analysed using a *HY-ALERTA 500* hydrogen sensor, as well as *X-STREAM* and *BINOS 100* instruments including a paramagnetic oxygen analyser and nondispersive infrared (NDIR) sensors for CO, CO_2_, and CH_4_. The density and heat capacity of the exhaust gas were estimated by adding the values of the individual gas components.

#### Specific heat capacity

The specific heat capacity values used in the calculations are listed in Table S1. The NIST Chemistry WebBook^31^ was used for copper, water, and the gas components, assuming a temperature of 300 K. For the steel pipes (mostly stainless steel) an approximate value of 0.5 J/gK was used and for the cells 1.0 J/gK was used (1.05 J/gK was known for the small prismatic cell at 50°C).

#### Accelerating rate calorimetry

The ARC chamber had a total volume of 0.6 L and the tests were conducted using heat-wait-seek operation mode with 5°C temperature steps. Test specifications are found in Table S2. A photo of the ARC chamber is found in Figure S6.
