# Supplementary material for: A calorimeter for analyzing ejected and non-ejected heat during Li-ion battery thermal runaway
Source: iScience. 2025 Jun 18;28(7):112941. doi: 10.1016/j.isci.2025.112941 (PMC12270710; doi:10.1016/j.isci.2025.112941)
Supplement: Document S1. Figures S1–S6, Tables S1 and S2 [file mmc1.pdf]

iScience, Volume *28*

Supplemental information

A calorimeter for analyzing ejected and non-ejected heat during Li-ion battery thermal runaway

Ola Willstrand, Mohit Pushp, Petra Andersson, and Daniel Brandell

Supplemental figures

Figure S1: Copper block test setup, related to Figure 1.

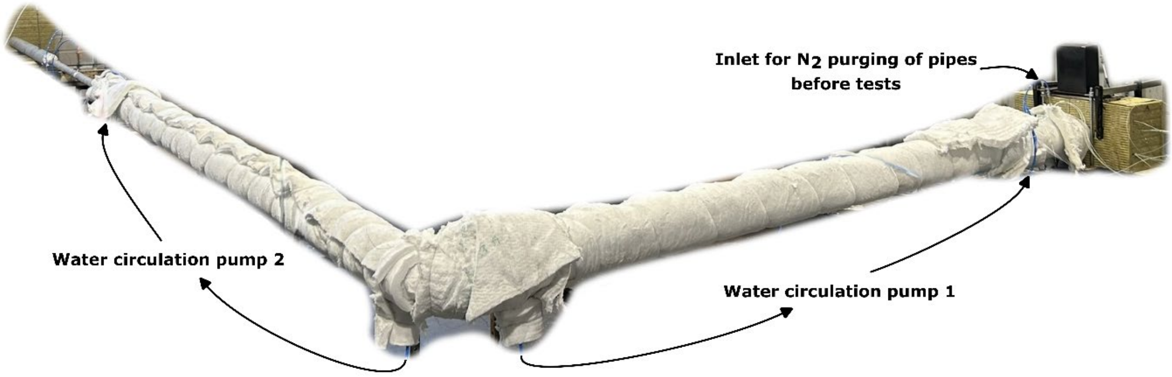

Figure S1. Photo of the copper block test setup.

Figure S2: CB1 and CB2 thermocouple positions, related to Figure 4.

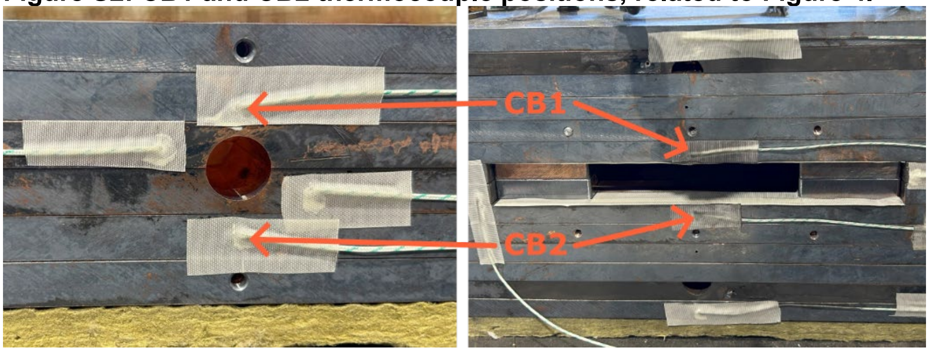

Figure S2. Thermocouple positions "CB1" and "CB2" in copper block adjusted for cylindrical cells (left) and prismatic cells (right). The distance between thermocouple and cell is 10 mm for the cylindrical cell configuration and 5 mm for the prismatic cell configuration.

Figure S3: Normal gas flow at different SOC levels, related to Figure 6.

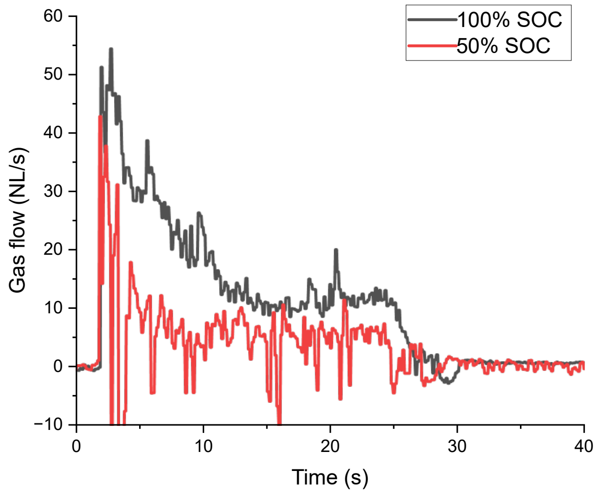

Figure S3. Normal gas flow for prismatic cell C at 100 % and 50 % SOC.

Figure S4: ARC test results, related to Figure 7.

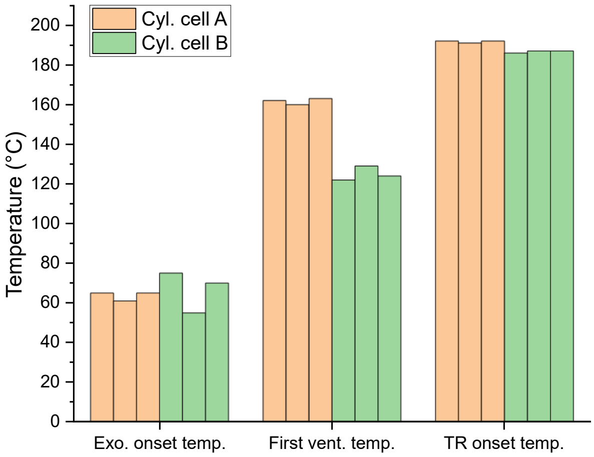

Figure S4. Exothermic onset temperature, temperature of first venting, and thermal runaway onset temperature for the 6 tests conducted in the ARC setup.

Figure S5: Example of corrected water temperatures, related to STAR methods.

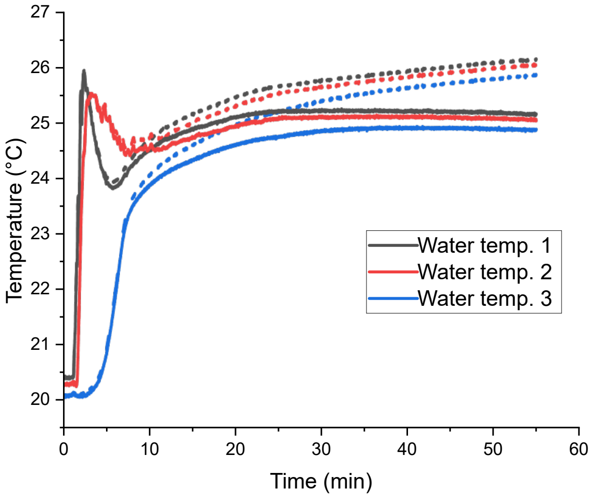

Figure S5. Original measurements (dotted lines) and corrected water temperatures (solid lines) where heat generation from the water circulation pump is subtracted.

Figure S6: ARC test chamber, related to STAR methods.

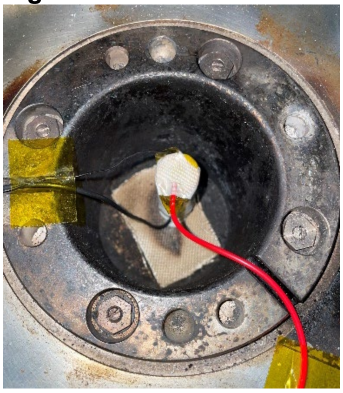

Figure S6. Photo of the ARC test chamber.

Supplemental tables

Table S1: Specific heat capacity values used in the calculations, related to STAR methods.

Component	Specific heat capacity (J/kgK)	Gases	Specific heat capacity (J/molK)
Copper block	385	Nitrogen	29
Water	4184	Hydrogen	29
Steel pipes	500	Carbon dioxide	37
Cells	1000	Carbon monoxide	29
		Methane	36

Table S2: Specifications of conducted ARC tests, related to STAR methods.

Cell type	Nr. of tests	SOC	Start temp.	Temp. step	Temp. rate sens.	Calc. temp. step	Data logging
Cyl. cell A	3	100%	50 °C	5 °C	0.02 °C	0.2 °C	1 °C/0.5 min
Cyl. cell B	3	100%	50 °C	5 °C	0.02 °C	0.2 °C	1 °C/0.5 min
